# Investigation of the genetic determinism of amino acid digestibility traits in Duroc×Landrace×Yorkshire crossbred pigs

**DOI:** 10.5713/ab.24.0765

**Published:** 2025-04-04

**Authors:** Yancan Wang, Xin Wang, Qiye Wang, Kang Xu, Zhi Li, Qin Zeng, Jianzhong Li, Yulong Yin, Huansheng Yang

**Affiliations:** 1 Hunan International Joint Laboratory of Animal Intestinal Ecology and Health, Laboratory of Animal Nutrition and Human Health, College of Life Sciences, Hunan Normal University, Changsha, China; 2 Key Laboratory of Agro-ecological Processes in Subtropical Region, Hunan Provincial Engineering Research Center of Healthy Livestock, Scientific Observing and Experimental Station of Animal Nutrition and Feed Science in South-Central, Ministry of Agriculture, Institute of Subtropical Agriculture, Chinese Academy of Sciences, Changsha, China; 3 Hunan Key Laboratory for Conservation and Utilization of Biological Resources in the Nanyue Mountainous Region, Hengyang Normal University, Hengyang, China

**Keywords:** Amino Acid Digestibility, Candidate Gene, Genome-wide Associated Study, Pig

## Abstract

**Objective:**

The objective of this study was to delineate the genetic architecture of ileal amino acid digestibility in Duroc×(Landrace×Yorkshire) hybrid (DLY) pigs through genome-wide association study (GWAS). The goal was to identify associated single nucleotide polymorphisms (SNPs) and candidate genes to inform precision breeding strategies for enhanced nutrient utilization and environmental sustainability.

**Methods:**

We conducted a GWAS on 600 DLY pigs to identify genetic markers associated with ileal amino acid digestibility, employing the GGP Porcine 50K SNP Chip and analyzing fifteen amino acid digestibility traits. GLM and FarmCPU-based GWAS approaches were utilized to detect SNP associations, followed by gene annotation to identify candidate genes near significant SNP loci.

**Results:**

We identified two SNPs, chr3:33019982 and Chr17:34715741, significantly associated with the digestibility of multiple amino acids. SNP chr3:33019982 was linked to threonine, leucine, histidine, proline, and arginine digestibility, while Chr17:34715741 was associated with arginine. Gene annotation revealed Trans-Golgi network vesicle protein 23 homolog A (TVP23A) and Synapse differentiation-induced gene I (SynDIG1) as potential candidates for amino acid metabolism in the terminal ileum.

**Conclusion:**

Our study identified key SNPs and genes linked to amino acid digestibility in DLY pigs, specifically highlighting the pleiotropic effects of the SNP WU_10.2_3_ 33019982 and the potential roles of TVP23A and SynDIG1 genes. These findings contribute to the molecular breeding of DLY pigs for improved amino acid utilization and provide insights into the genetic control of this trait.

## INTRODUCTION

In recent decades, pork has constituted a substantial proportion of global meat production to meet the increasing demands of human consumption. In livestock systems, proteins feeds are among the most expensive and limiting ingredients in diet formulations, and the supply of protein source in feed has been constrained by the international environment and other conditions, such as political regulations, climate and natural disasters, and social factors [1]. Swine account for approximately 42% of the total feed production and contribute 20% of animal proteins [2]. However, high-protein feed is associated with increased urea excretion, ammonia production, and a higher risk of intestinal health damage in pigs [3]. The nutritional requirements for crude protein are primarily determined by amino acids intake [4]. Variability in nutritional demand and amino acids digestion utilization among individuals within the same pig herd poses significant challenges to the accurate supply of feed nutrition [5]. Therefore, elucidating the genetic mechanism of nutrition digestion and utilization in pigs and developing stable, efficient pig breeds with enhanced nutrition efficiency are effective strategies for precise nutrition supply. Currently, several molecular markers that shed light on the genetic basis of pig physiological traits and morphological traits have been identified through genome-wide association study (GWAS), including those related to meat quality [6], body shape [7], coat color [8], disease resistance [9,10], carcass length [11], and the number of thoracolumbar vertebrae [12]. However, there remain inadequacies and challenges in elucidating the complex biological mechanisms of digestion and absorption in pigs; while LI et al [13] identified several genes associated with fiber apparent digestibility traits and single nucleotide polymorphisms (SNPs) to analyze the genetic mechanism of roughage tolerance in Su-Huai pigs, there are scant reports on quantitative trait locus (QTL) or SNPs related to amino acid digestibility traits in the terminal ileum of pigs. Therefore, identifying genes associated with the digestion, absorption and deposition of amino acids is crucial for enhancing nutritional utilization, reduce environmental pollution and achieve precise nutrition in pigs.

With the aid of high-density SNPs across the porcine genome, GWAS was utilized to identify genes associated with amino acid digestibility traits in pigs. A recently developed GWAS model, named the fixed and random model Circulating Probability Unification (FarmCPU) [14], has been widely applied for detecting QTLs for economically important traits [15]. The major feature of FarmCPU is to account for the effects of other markers by including multiple markers simultaneously as covariates, avoids model overfitting, and maintains false positives simultaneously for an efficient computation [14].

The Duroc×(Landrace×Yorkshire) hybrid pigs (DLY) are the most popular commercial pigs utilized in the Chinese pig industry. Furthermore, pigs serve as a valuable animal model for studying the genetic basis of human diseases due to their numerous physiological and phenotypic similarities with humans, including those related to digestive tract diseases. Human digestion, a multi-step and multi-compartmental process, is essential for human health, and in vivo approaches in humans are constrained by evident ethical, regulatory, and technical constraints [16]. To our knowledge, this study represents the first genome-wide investigation of ileal terminal amino acid digestibility traits in pigs, aiming to provide a foundation for elucidating the genetic control of digestive and absorption characteristics in animals.

## MATERIALS AND METHODS

The experimental design and procedures in this study were reviewed and approved by the Animal Care and Use Committee of Hunan Normal University (Approval number: 2019-208), Changsha, Hunan, P.R. China.

### Experimental animals

Three-way crossbred DLY pigs intercrossed by Duroc boars and (Landrace×Yorkshire) sows were used in this study to conduct genetic analyses for amino acid digestibility traits. The experimental animals used in this study consisted of 600 DLY boars born in 2019 and raised in the same farm of Wen’s Foodstuffs Group Co., Ltd. (Guangdong, China). All pigs were fed with the same diets and had unlimited access to drinking water. They were housed in the same barn, with approximately 12 pigs in each pen, and raised under the same management conditions. They were slaughtered one day at the age of 210±3 days in a commercial abattoir in Hunan Changzhutan Grandline Pig Trading Market Co., Ltd.

### Sample treatment and collection

The pigs were slaughtered by electrical stunning (1.3 A for at least 3 s to apply 240 V stunning voltage), and then they were exsanguinated, scalded, skinned, eviscerated, and split down from the midline according to standard commercial procedures. The boundaries between the jejunum, cecum, and colon were ligatured to prevent chyme flow into other parts of the intestine. Then, ileal digesta samples were collected in 50 mL centrifuge tubes and immediately frozen in liquid nitrogen and stored at −80°C until required for amino acids digestibility analysis. Intestinal tissues from the middle part of the ileum were collected (approximately, 6 cm of each tissue) and immediately frozen in liquid nitrogen and stored at −80°C until the use for DNA extraction and mRNA analysis.

### Digestibility study and chemical analysis

Before analyses, ileal digesta samples were lyophilized using a freeze drier (Labconco Freezone 2.5L Freeze Dry System; Marshall Scientific, Hampton, NH), and then, the test diets and freeze-dried ileal digesta samples were ground through a 1-mm screen. The dry matter (DM) content of the experimental diets was analyzed by drying the samples at 135°C for 2 h. The acid-insoluble ash analysis method was based on those described by Keulen and Young [17]. Briefly, feed and ileal digesta samples were weighed at 2.0±0.001 g in triplicate in a conical flask, and then 100 mL of 4N HCl was added and boiled on an electric heating plate for 30 min. A condenser was attached to the conical flask to prevent the loss of HCl. The thermal hydrolysate was then filtered with an ash-free filter paper (Whatman No. 541), and the residue was washed with acid-free hot double-distilled water at 85°C to 100°C. Subsequently, the filter paper and residue were transferred to a tared crucible and dried for 48 h at 103°C and ashed for 4 h in a high-temperature furnace at 600°C. After ashing, the crucible was cooled to room temperature in a desiccator and weighed. The acid-insoluble ash content was calculated using the following equation [18]:


(1) Acid-insolubleash (%)=(Wf-We)/Ws×100

where Wf represents the weight of crucible with ash, We represents the weight of empty crucible, and Ws represents the weight of the sample DM.

Feed and ileal digesta amino acid concentrations were analyzed using Hitachi Amino Acid Analyzer (L-8900, Hitachi High-Tech Science Corporation, Tokyo, Japan). Prior to analysis, the samples were weighted at 1.0±0.001 g (in triplicate) in a hydrolysis tube, and then 10 mL of 6 N HCl was added, sealed by an alcohol blast burner, and hydrolyzed at 110°C for 24 h. After that, the hydrolysis tube was cooled to room temperature, then the tube was opened, and the hydrolysate was filtered into a 50-mL colorimetric tube via Whatman No. 1 paper that was rinsed with H_2_O for three times, and then H_2_O was used to constant volume. After mixing, a 1-mL solution was transferred to a 10 mL colorimetric tube and dried in a water bath at 65°C. The dry hydrolysate was dissolved in 2 mL 0.02N HCl and filtered through a 0.22-μm membrane. Then, the amino acid contents were determined using an automatic amino acid analyzer.

The apparent ileal digestibility (AID) of amino acid was calculated using the following equation with internal acid-insoluble ash as a marker [18]:


(2) $$\text{AID }(\%)=(1-[{ash}_{diets}/{ash}_{digesta}]\times [AA_{digesta}/AA_{diets}])\times 100$$

where *ash_diets_* and *ash_digesta_* represent the internal acid-insoluble ash concentrations in the diets and ileal digesta from pigs, respectively (g per kg DM), and AA diets and AA digesta represent the AA concentrations in the diets and ileal digesta from pigs, respectively (g per kg DM).

### Statistical analysis of phenotypic data

IBM SPSS 20.0 (IBM Corp, Armonk, NY, USA) was employed to produce descriptive statistics, including the number, minimum, maximum, mean, standard deviation, standard error, and coefficient of variation, of fifteen ileal terminal amino acid digestibility traits for pig accessions. The hist () function of R software is used to visualize the phenotypic data as a frequency distribution histogram. If the phenotypic data of traits do not conform to normal distribution, the R package “FRGEpistasis” is employed to normalize them for GWAS analysis. Phenotypic parameters analysis was conducted using SPSS software and visualized using the R package “ggplot2”. The genetic correlations for fifteen amino acid digestibility of terminal ileum traits were estimated using GCTA software [19]. A genetic analysis was undertaken to estimate the genetic variance of terminal ileal amino acid digestibility as well as their genetic correlations with other traits. Heritability estimates were qualified as low below 0.20, moderate from 0.20 to 0.40 and high above 0.40. Genetic correlations were considered low for absolute values between 0.00 and 0.20, moderate between 0.20 and 0.50 and high above 0.50.

### Genotyping and quality control

Genomic DNA was extracted from ear tissues using an animal tissue DNA extraction kit (Generay Biotech Co., Ltd., Shanghai, China) following the manufacturer’s protocol. DNA quality was detected using a NanoDrop ND-1000 (Peqlab Biotechnology, London, UK) and agarose gel electrophoresis. The DNA concentration of the samples was adjusted to 50 ng/μl. Samples were genotyped with the GGP Porcine 50 K SNP Chip (Neogen, Lincoln, NE, USA). Quality control was carried out using PLINK v1.07 [20] software. SNPs with call rates lower than 95%, ambiguous locations, and minor allele frequencies less than 0.01 were discarded. SNPs that failed the Hardy–Weinberg equilibrium test (p<0.001) and unmapped were also removed.

### Population structure and association analyses

Principal component analysis (PCA) was performed using the SNP dataset through rMVP to assess the potential population stratification prior to conducting the GWAS [21]. The SNP chip genotype data and fifteen traits phenotypic data of pigs were analyzed by genome-wide efficient mixed-model analysis (GEMMA) software [22]. Using R packet rMVP [21] includes two models: general linear model (GLM), and FarmCPU [23].

#### General linear model-based Sgenome-wide association study

The fifteen traits were analyzed using the general linear mixed (GLM) model fitted in GEMMA software [22], one trait at a time. The statistical linear mixed model is described as follows:


(3) Y=Wα+Xβ+μ+ɛ

where *Y* is an n×1 vector of phenotypes in the DLY pig population; α is a vector of the corresponding parameters including the intercept, sex, body weight (not included for BW), and the top five eigenvectors obtained prior to this analysis using the GCTA software [24]; *W* is the incidence matrix of the appropriate dimension for the fixed effects; *β* is the effect of the marker; *X* is an n×1 vector of marker genotypes; *u* ~ MVN (0, A σ^2^_a_) is an n×1 vector of animal residual additive genetic effect without accounting for the fitted SNP effects with *A* being the genomic relationship matrix estimated; and *ɛ* ~ MVN (0, I σ^2^_e_) is the vector of residual errors, where I_n_ is an n×n identity matrix.

#### FarmCPU-based genome-wide association study

The GAPIT (version 3.0) R package [23] was used to conduct FarmCPU-based GWAS. All parameters were set as default. Briefly, the FarmCPU model consists of two parts: the fixed-effect model (FEM) and the random-effect model, which is evaluated iteratively. The effects in the FEM include the top five principal components, sex, and pseudo quantitative trait nucleotides (QTNs) as [14] as follows:


(4) $$Y=Pb_{p}+M_{t}B_{t}+s_{j}d_{j}+e$$

where *y* is a vector of phenotypes of the analyzed trait; *b_p_* is a vector of fixed effects including top five principal components calculated by GAPIT, sex, and Body Weight (not included for BW); *b_t_* is a vector of the fixed effects for the pseudo QTNs; *P* and *M_t_* are the corresponding incidence matrices for *b_p_* and *b_t_*, respectively; *d_j_* is the effect of the *j-th* candidate SNP; *s_j_* is the genotype for the *j-th* candidate SNP; and *e* is a vector of the residuals.

The genetic relationship matrix and principal components are added to the model as covariables to reduce the influence of kinship and population structure. The genome-wide significant level α<0.05; GWAS results were corrected by Bonferroni (0.05/N, N was the number of markers) to reduce the false positive. The whole genome significance threshold was p<1.75×10^−6^. To reduce the probability of false positives, this study used the R language qqman software package to plot the quantile–quantile (Q-Q) plots to assess the influence of potential population stratification on GWAS [25].

### Gene annotation and screening of candidate genes

GWAS analysis was performed to obtain SNP significantly associated with the target traits, and the pig reference genome database at Ensembl (https://asia.ensembl.org/index.html) was used to identify the chromosomal and physical locations of these significant SNP and to identify related genes within 100 kb upstream and downstream of the SNP. Information from the NCBI (https://www.ncbi.nlm.nih.gov) and *Ensembl genome databases* was integrated for gene function annotation and to identify candidate gene associated with the target traits.

## RESULTS

### Phenotype description and correlation

**Figure 1:**
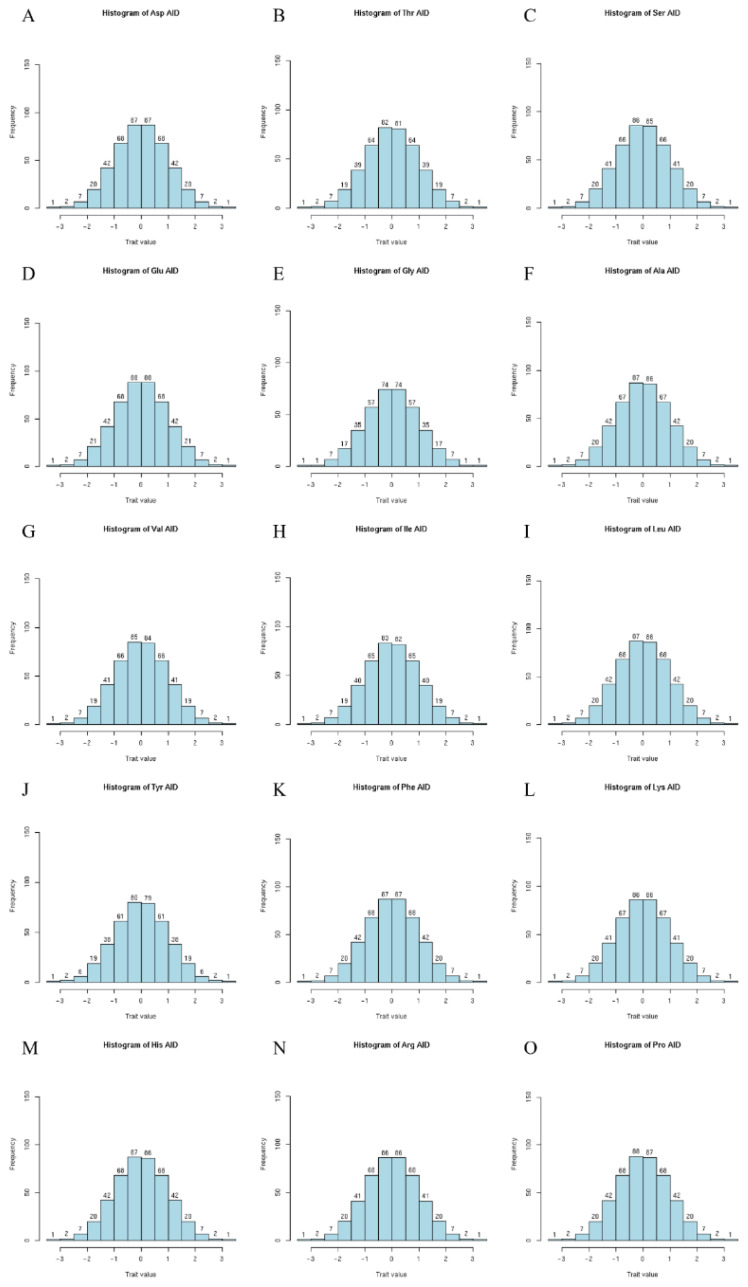
Normalized frequency distribution histogram of DLY pigs. DLY, Duroc×(Landrace×Yorkshire) hybrid pigs.

The statistical information on the fifteen traits is shown in Table 1. There is a big difference between the maximum and minimum values of the data. The variation coefficient of characters ranges from 15.22% to 24.16%. The results, therefore, indicated that three-way crossbred DLY pig populations had a large variation for fifteen amino acid digestibility of terminal ileum traits. The frequency distributions of the traits are shown in Figure 1. After normalization, all the fifteen traits appear to conform to the normal distribution. The phenotypic correlation coefficients for the fifteen amino acid digestibility traits are shown in Table 2. The results revealed that there was a significant positive correlation among fifteen terminal ileal amino acid digestibility (r>0.74, p<0.01).

### Variance components for terminal ileal amino acid digestibility

Heritability, genetic and phenotypic variances of the fifteen terminal ileal amino acid digestibility are presented in Table 3. Apart from Gly-AID (about 0.10±0.11), the heritability of amino acid digestibility rates ranges from 0.17 to 0.34, indicating low to moderate genetic influence. The estimated values of phenotypic variation of all traits are similar. Table 4 presents the genetic correlation estimates for all amino acid traits at the terminal ileum. There are moderate to high levels of genetic correlation among most of the amino acid digestibility rates, with some approaching values close to 1.00. This suggests a striking similarity in the underlying genetic determinants among certain amino acid digestibility rates.

### Quality control and population structure

**Figure 2:**
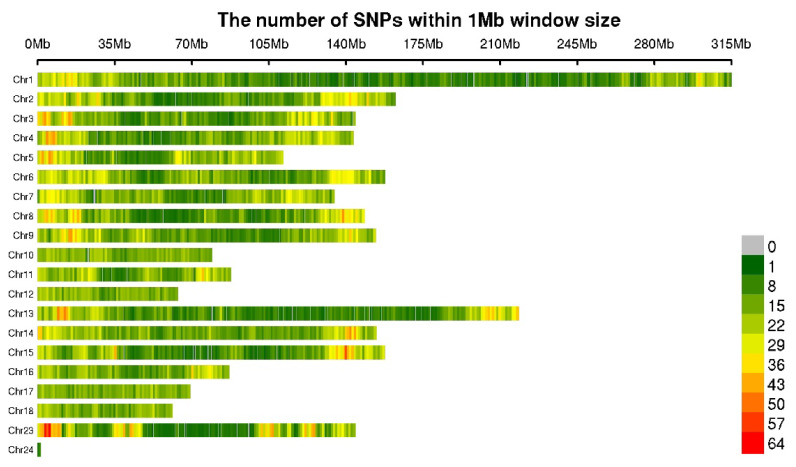
The filtered SNP density distributions on Sscrofa chromosomes. The horizontal axis (x-axis) shows the chromosome length (Mb). Colour index indicates the number of labels. SNP, single nucleotide polymorphism.

**Figure 3:**
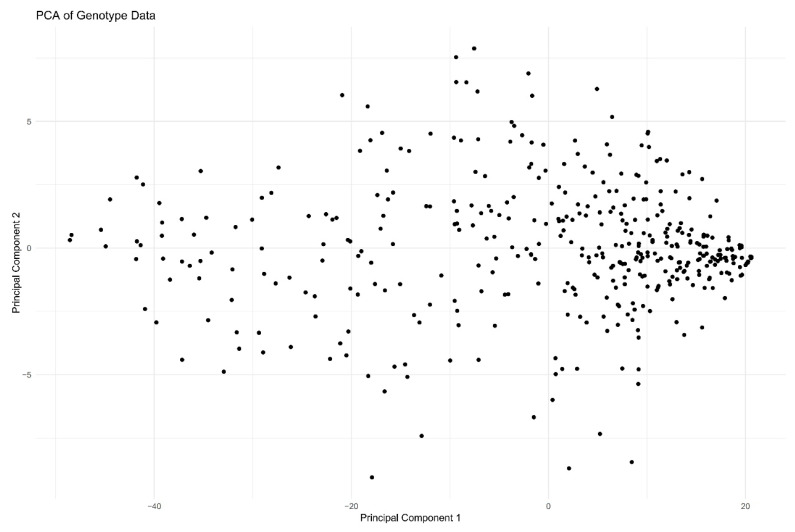
Plot of the principal components of DLY pigs. PCA, principal component analysis; DLY, Duroc×(Landrace×Yorkshire) hybrid pigs.

After analysis of microarray data and individual data according to quality control criteria, 41430 SNPs and 600 individuals were obtained for genome-wide association analysis studies. These SNPs were distributed on 18 autosomes and X/Y sex chromosomes, and their distribution and mean SNP distances are shown in Table 5 and Figure 2. In the principal component analysis of Figure 3, the scatter plot reveals no distinct sub-groupings among the samples, indicating a similar genetic background. Hence, it is conducive to subsequent GWAS analysis.

### Association analyses

We used the R package rMVP for genome-wide association analysis [21], containing two models: GLM and FarmCPU. the kinship matrix and principal components were added to the model as covariates for correction to reduce the effect of kinship and population structure. The threshold of significance at the genome-wide level was 0.05/markers number. GWAS analysis was performed for fifteen phenotypic traits in pigs, and the results are shown in Table 6. Five traits were finally localized to significant loci, Arg_AID (GLM, FarmCPU model), Asp_AID (GLM model), His_AID (GLM, FarmCPU model), Leu_AID (GLM, FarmCPU model) The other phenotypes did not locate significant loci. We calculated the λ values based on the p-values for each trait, which reflect the extent of inflation for each trait. These λ values range from 65.97 to 106.63, indicating varying degrees of inflation.

**Figure 4:**
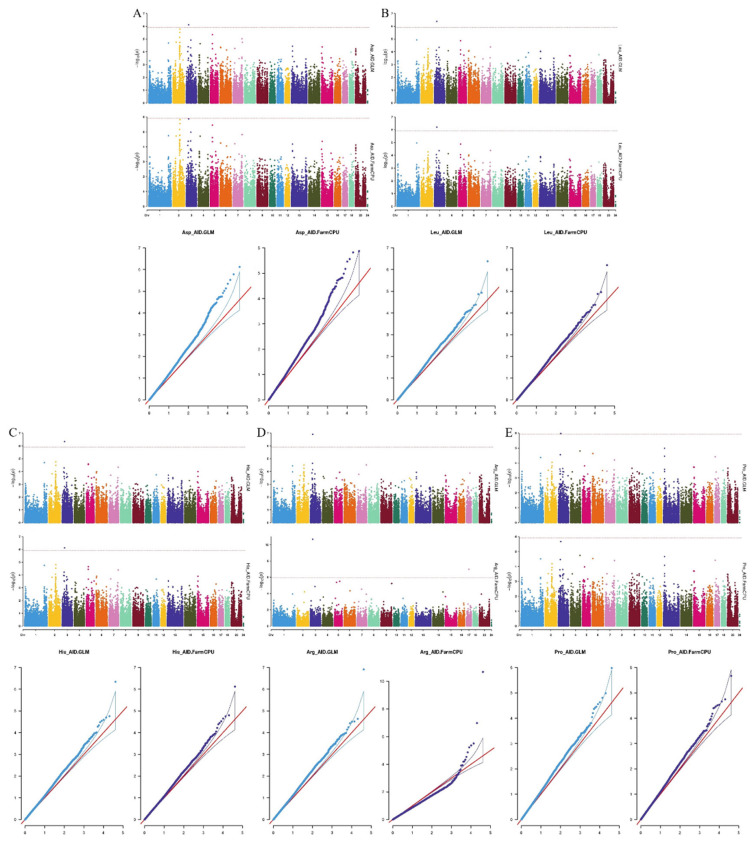
Manhattan plots and quantile-quantile (Q-Q) plots for amino acid digestibility of terminal ileum using GLM (up) and FarmCPU (down). (A) Asp_AID, (B) Leu_AID, (C) His_AID, (D) Arg_AID, (E) Pro_AID. Negative log^10^ p-values of the filtered high-quality SNPs were plotted against their genomic positions. The dashed lines of orange correspond to the Bonferroni-corrected thresholds of p = 1.75×10^−6^. The x-axis represents the chromosomes, and the y-axis represents the −log10 (p-value). Q-Q plots show the observed versus expected negative log 10 p-values. Asp_AID, aspartic digestibility of terminal ileum; Leu_AID, leucine digestibility of terminal ileum; His_AID, histidine digestibility of terminal ileum; Arg_AID, argnine digestibility of terminal ileum; Pro_AID, proline digestibility of terminal ileum; SNP, single nucleotide polymorphism.

Quantile-quantile plots of all traits were drawn since population stratification could have an impact on GWAS. We found that the observed −log_10_ *p* values of the GLM and FarmCPU were fairly close to the expected −log_10_ *p* values. The results showed that the GLM and FarmCPU well controlled the research’s false positives. Q-Q plot of each amino acid digestibility trait was shown following the manhattan plot of the corresponding traits (Figure 4). In total, 9 SNPs were identified as significant (p<1.75×10^−6^) for the traits investigated using GLM and FarmCPU (Table 5), but eight of the nine SNPs were the same. One SNP was significantly associated with the digestibility of aspartate, leucine, histidine, arginine, and proline (Asp_AID, Leu_AID, His_AID, Arg_AID, and Pro_AID) at the terminal ileum, and the locus WU_10.2_3_ 33019982 was located on chromosome 3. The other SNP was significantly associated with Arg_AID and is located on chromosome 17.

### Identification of candidate genes

In this study, a total of 2 genes located within 100 kb upstream and downstream of these significant SNPs were considered potential candidate genes. Based on the porcine reference genome database (*Sscrofa 10.2*) on the Ensembl website, the genes were selected from the NCBI website for functional annotation, as shown in Table 7.

## DISCUSSION

In pig production, the cost of feeding is usually measured by computing the feed conversion ratio, which is a ratio between two traits of interest in most breeding schemes (feed intake and growth rate) [26]. Breeding varieties with high feed conversion rate will improve production performance by improving digestibility. Crews [27] showed that the genes that regulate growth may also be involved in the regulation of feed conversion, which we suspect may be caused indirectly by affecting digestibility. It is well known that high feed intake should be established on the basis of strong digestibility, otherwise it will lead to the decrease of feed utilization rate and the increase of feed coefficient. In fact, there is a negative correlation between digestibility and feed intake, and the increase of feed intake will lead to the decrease of animal digestibility [28]. Vigors et al [29] found that ileal apparent digestibility, total energy, nitrogen and DM digestibility of RFI pigs with low residual feed intake increased significantly, and the expression levels of intestinal enzymes and transporters (SGLT1, GLUT2, SI, etc.) involved in digestion and absorption were significantly increased. Unfortunately, this paper does not record feed intake, daily gain and other production performance data, which is insufficient.

However, we must realize that effectively improving the feed intake and digestibility of nutrients (especially protein) of medium and large pigs is one of the important ways to improve the economic benefits of raising pigs. Ileal terminal amino acid digestibility is usually used to indicate the effect of feed protein and amino acids [30]. Therefore, screening the excellent varieties with high amino acid digestibility at the terminal ileum can not only improve the production performance of pigs, but also avoid feed waste and inefficient utilization, and improve the economic benefits of pig breeding.

Genome-wide association studies provide an opportunity to dissect the genetic architecture of complex traits by leveraging linkage disequilibrium between the causative mutations and common SNP markers in pigs [31]. We performed two model-based GWAS on ileal terminal amino acid digestibility traits in a DLY pig population, detecting a set of trait-related SNPs, and then based on these SNPs, candidate genes were annotated.

Each of these methods has its unique features and applications, catering to different aspects of genetic analysis. GCTA is primarily used for estimating the heritability of complex traits attributed to all SNPs across the genome [24]. It can also perform genomic-relatedness-based restricted maximum likelihood analysis to estimate the proportion of phenotypic variance explained by all SNPs. The findings presented in this study confirmed that amino acid digestibility of terminal ileum are heritable traits in pigs and that they are genetically correlated to each other. Apart from Gly-AID, the heritability of amino acid digestibility rates ranges from 0.17 to 0.34, indicating low to moderate genetic influence, which is consistent with the variation in nitrogen digestibility efficiency estimated by Déru et al [32] in pigs, this also implies that genetic factors play a significant role in explaining the variability of digestibility rates, but they are also influenced by other factors such as environment, diet, batch effects, and other non-genetic factors. For example, it has been found that when birds were fed a diet that is more digestible, their heritability is much lower [33]. Feed and genetics played a role in explaining the variation in digestive efficiency, yet other key factors remained to be identified. While the batch effect was noticeable, its impact was constrained in our study due to the standardized feed resources. Additionally, the pen effect likely encompasses the influence of the birth herd, which shares an initial environment and gut microbiota acquired early in life. These factors could affect later digestive efficiency in pigs, given the significant role of gut microbiota in nutrient digestibility in this species [34]. Based on a comprehensive dataset set reflecting a commercial pig population, heritabilities estimated also affirm that digestive efficiency is indeed an inheritable characteristic, as suggested by Noblet et al [35]. In this study, a substantial portion of amino acid digestibility rates at the terminal ileum exhibit moderate to high levels of genetic correlation, indicating significant genetic similarity among them. This may be attributed to shared metabolic pathways, common enzyme systems among amino acids, as well as individual genetic backgrounds and intestinal microbiota composition. Additionally, this may also reflect the influence of familial environment, genetic background, and shared lifestyle factors on the formation of such genetic correlations, which could be of significant relevance to understanding the genetic basis of nutritional metabolism and related disorders.

In our GWAS, two models were employed: the generalized linear model (GLM) [36] was utilized without accounting for random effects in the association analysis to identify relationships between traits and SNPs. FarmCPU, which dynamically updating fixed effects, overcomes the limitations of GLM and provides a robust approach for detecting associations while controlling for background genetic noise, making it particularly suitable for crop and livestock research [14]. Consequently, this enabled us to leverage the strengths of both models to accurately identify and analyze genetic factors associated with amino acid digestion characteristics while reducing potential biases induced by population structure and relatedness, thus enhancing the accuracy and statistical power of the association signals.

Through the joint screening of the two models, we observed that the first SNP (WU_10.2_3_33019982) is significantly associated with the digestibility of arginine, aspartic, leucine, histidine and proline (Asp_AID, Leu_AID, His_AID and Pro_AID) among the amino acid traits investigated in the terminal ileum, while the second SNP (ALGA0094389) is only significantly associated with arginine digestibility. This observation may be related to the phenotypic and genetic correlations among these traits. By conducting phenotypic and genetic correlation analyses among all amino acid digestibility traits, we found that these traits exhibit moderate to high correlation coefficients, suggesting that they may share common metabolic pathways or enzyme systems in their physiological functions. For example, the digestion of certain amino acids may depend on the same enzymes or transporters, leading to genetic correlations among these traits. This pleiotropic effect indicates that certain genes may play important roles in the digestion of multiple amino acids. On the other hand, the second SNP (ALGA0094389) is only significantly associated with arginine digestibility, which may indicate the unique physiological function of arginine. Arginine plays a crucial role in protein synthesis and energy metabolism, and its digestibility may be regulated by specific genes. This specificity may reflect the unique position of arginine in metabolic processes. To further validate the biological significance of these associations, we plan to conduct validation in independent cohorts and explore the specific effects of these SNPs on amino acid digestibility through experimental approaches, such as gene expression analysis. These validations will help confirm the functional roles of these SNPs in the amino acid digestion process. In conclusion, we believe that the associations between these SNPs and amino acid digestibility traits are biologically plausible, and the correlations among these traits may reflect their common mechanisms in metabolic processes. These findings provide an important foundation for future research and offer new insights into the genetic regulation of amino acid digestion. The gene adjacent to WU_10.2_3_ 33019982 was *TVP23A* (Gene ID: 780776), while the gene located nearALGA0094389 was Synapse differentiation-induced gene I (*SynDIG1*).

*TVP23A* encodes the trans-Golgi network vesicle protein 23 homolog A (formerly known as FAM18A), which is involved in intracellular vesicular transport. Although *TVP23A* has been reported as a candidate gene for late-onset Parkinson’s disease [37], its role in amino acid digestibility has not been elucidated. According to the Human Protein Atlas1 [38], *TVP23A* is predominantly expressed in macrophages among the 29 identified cell-types [NCBI/GEO accession number GSE111976]. In macrophages, amino acids play crucial functional and regulatory roles [39]. Glutamine metabolism has been associated with anti-inflammatory polarization programs, while serine metabolism is implicated in the regulation of IL-1β production [40]. Furthermore, amino acids also serve as precursors for the production of effector molecules in macrophages. Lipopolysaccharide(+IFNγ)-induced macrophages primarily convert arginine into nitric oxide (NO) via inducible NO synthase (iNOS), whereas IL-4-stimulated macrophages predominantly metabolize arginine into ornithine and polyamines [41]. This suggests that *TVP23A* may influence the amino acid digestibility trait of the terminal ileum by modulating the effector factors produced by amino acids in macrophages.

*SynDIG1* is identified as an α-amino-3-hydroxy-5-methylisoxazole-4-propionic acid subtype glutamate receptor (AMPAR)-interacting transmembrane protein with a large intracell ular N-terminal region and a single transmembrane domain that could regulate excitatory synapse development via AMPAR content [42]. *SynDIG1* was reported to play a central role in muscle, metabolism, and neurobiology [43]. For example, *SynDIG1* has been found to be associated with depressive symptoms [44] and can be used as a biomolecule to predict thyroid cancer [45]. In animal research, *SynDIG1* has been reported as a factor affecting the final weight and backfat thickness of Landrace pigs [46], and it is also a candidate gene affecting body size traits in Simmental beef cattle [47]. Therefore, we suspect that *SynDIG1* affects its growth performance and body size traits by enhancing the amino acid digestibility of the terminal ileum.

Unfortunately, only two significant SNPs were identified, which may be attributed to the relatively small sample size of 600, which limits the detection of SNPs, particularly those with smaller effect sizes. Additionally, the use of two models for GWAS screening with stringent statistical criteria ensures that only SNPs with a strong association with the trait are selected, thereby minimizing potential false positives. Furthermore, the complexity of ileal amino acid digestibility traits suggests that they may be influenced by multiple genes, each with potentially minor effects. In such cases, GWAS may detect only a subset of genes with larger effects, potentially overlooking others with minor effects. Therefore, this does not indicate a failure of GWAS but rather reflects the complexity and challenges inherent in detecting ileal amino acid digestibility traits.

## CONCLUSION

In this study, we identified two SNPs associated with five amino acid digestibility traits of the terminal ileum in DLY pigs using GLM and FarmCPU-based GWAS. Subsequently, we identified two genes associated with amino acid digestibility traits in pigs. WU_10.2_3_33019982 was the only SNP affecting more than three traits and exhibiting pleiotropic effects on Asp_AID, Leu_AID, His_AID, Arg_AID, and Pro_AID. Specifically, the *TVP23A* gene is proposed as a strong candidate for amino acid digestibility traits. Additionally, the candidate genes *SynDIG1* located near the significant SNP site was identified through gene annotation and may be associated with Arg_AID. We anticipate that our results will provide a comprehensive understanding of the amino acid digestibility trait, which has not been sufficiently studied in pigs. Overall, this study not only advances molecular breeding for amino acid digestibility trait in DLY pig but also enhances our understanding of the poorly characterized genes controlling amino acid digestibility in pigs.

## Figures and Tables

**Table 1:** Descriptive statistics of phenotypic data

Traits (%)	N	Min	Max	Mean	SD	SE	CV (%)
Asp_AID	421	45.78	99.81	85.99	13.48	0.66	15.68
Thr_AID	400	25.50	99.59	77.99	18.84	0.94	24.16
Ser_AID	420	32.39	99.74	80.50	17.28	0.84	21.47
Glu_AID	430	47.75	99.80	86.09	13.10	0.63	15.22
Gly_AID	357	34.20	99.86	80.06	16.78	0.89	20.96
Ala_AID	421	29.63	99.68	80.69	17.47	0.85	21.65
Val_AID	410	25.02	99.72	79.55	18.62	0.92	23.40
Ile_AID	404	28.22	99.66	80.17	18.52	0.92	23.10
Leu_AID	423	35.18	99.76	82.89	16.49	0.80	19.89
Tyr_AID	383	28.22	99.62	79.32	17.92	0.92	22.59
Phe_AID	422	39.09	99.79	84.38	15.06	0.73	17.85
Lys_AID	414	28.65	99.91	81.31	17.70	0.87	21.77
His_AID	422	36.92	99.97	83.45	15.79	0.77	18.92
Arg_AID	420	33.10	99.98	83.18	16.92	0.83	20.34
Pro_AID	427	46.32	99.81	85.36	13.40	0.65	15.70

N, number of accessions; Min, minimum; Max, Maximum; SD, standard deviation; 6)SE, standard error; CV, coefficient of variation; Asp_AID, aspartic digestibility of terminal ileum; Thr_AID, threonine digestibility of terminal ileum; Ser_AID, serine digestibility of terminal ileum; Glu_AID, glutamic acid digestibility of terminal ileum; Gly_AID, glycine digestibility of terminal ileum; Ala_AID, alanine digestibility of terminal ileum; Val_AID, valine digestibility of terminal ileum; Ile_AID, isoleucine digestibility of terminal ileum; Leu_AID, leucine digestibility of terminal ileum; Tyr_AID, tyrosine digestibility of terminal ileum; Phe_AID, phenylalanine digestibility of terminal ileum; Lys_AID, lysine digestibility of terminal ileum; His_AID, histidine digestibility of terminal ileum; Arg_AID, argnine digestibility of terminal ileum; Pro_AID, proline digestibility of terminal ileum.

**Table 2:** Phenotypic correlation coefficients among fifteen terminal ileal amino acid digestibility traits in crossbred pigs

Trait (%)	Asp_AID (%)	Thr_AID (%)	Ser_AID (%	Glu_AID (%)	Gly_AID (%)	Ala_AID (%)	Val_AID (%)	Ile_AID (%)	Leu_AID (%)	Tyr_AID (%)	Phe_AID (%)	Lys_AID (%)	His_AID (%)	Arg_AID (%)
Thr_AID	0.976**													
Ser_AID	0.974**	0.996**												
Glu_AID	0.988**	0.992**	0.988**											
Gly_AID	0.709**	0.778**	0.752**	0.751**										
Ala_AID	0.986**	0.994**	0.991**	0.998**	0.752**									
Val_AID	0.988**	0.990**	0.986**	0.997**	0.751**	0.996**								
Ile_AID	0.984**	0.985**	0.978**	0.991**	0.767**	0.990**	0.994**							
Leu_AID	0.988**	0.989**	0.984**	0.995**	0.740**	0.994**	0.998**	0.996**						
Tyr_AID	0.959**	0.989**	0.982**	0.984**	0.796**	0.984**	0.979**	0.977**	0.982**					
Phe_AID	0.987**	0.988**	0.983**	0.995**	0.745**	0.993**	0.995**	0.991**	0.997**	0.985**				
Lys_AID	0.988**	0.981**	0.976**	0.992**	0.740**	0.991**	0.992**	0.989**	0.994**	0.977**	0.996**			
His_AID	0.985**	0.990**	0.985**	0.995**	0.751**	0.995**	0.994**	0.990**	0.996**	0.986**	0.996**	0.994**		
Arg_AID	0.960**	0.976**	0.963**	0.970**	0.749**	0.968**	0.967**	0.966**	0.974**	0.969**	0.973**	0.971**	0.976**	
Pro_AID	0.982**	0.990**	0.989**	0.993**	0.741**	0.993**	0.992**	0.984**	0.989**	0.972**	0.988**	0.981**	0.986**	0.956**

** The correlation is significant (p<0.01).

Asp_AID, aspartic digestibility of terminal ileum; Thr_AID, threonine digestibility of terminal ileum; Ser_AID, serine digestibility of terminal ileum; Glu_AID, glutamic acid digestibility of terminal ileum; Gly_AID, glycine digestibility of terminal ileum; Ala_AID, alanine digestibility of terminal ileum; Val_AID, valine digestibility of terminal ileum; Ile_AID, isoleucine digestibility of terminal ileum; Leu_AID, leucine digestibility of terminal ileum; Tyr_AID, tyrosine digestibility of terminal ileum; Phe_AID, phenylalanine digestibility of terminal ileum; Lys_AID, lysine digestibility of terminal ileum; His_AID, histidine digestibility of terminal ileum; Arg_AID, argnine digestibility of terminal ileum; Pro_AID, proline digestibility of terminal ileum.

**Table 3:** Heritability (h^2^), genetic and phenotypic variances of ileal terminal amino acid digestibility, for Duroc×(Landrace×Yorkshire) hybrid pigs, along with their standard error (SE)

Traits (%)	h^2^ (SE)	Genetic variance (SE)	Phenotypic variance (SE)
Asp_AID	0.31 (0.11)	0.32 (0.12)	1.04 (0.16)
Thr_AID	0.19 (0.11)	0.19 (0.12)	1.01 (0.16)
Ser_AID	0.23 (0.10)	0.23 (0.11)	1.03 (0.15)
Glu_AID	0.17 (0.10)	0.18 (0.10)	1.03 (0.14)
Gly_AID	0.10 (0.11)	0.10 (0.12)	1.00 (0.17)
Ala_AID	0.20 (0.10)	0.20 (0.11)	1.03 (0.15)
Val_AID	0.22 (0.11)	0.23 (0.11)	1.03 (0.16)
Ile_AID	0.30 (0.12)	0.31 (0.13)	1.03 (0.17)
Leu_AID	0.21 (0.10)	0.21 (0.11)	1.03 (0.15)
Tyr_AID	0.34 (0.12)	0.35 (0.13)	1.02 (0.17)
Phe_AID	0.22 (0.10)	0.23 (0.11)	1.03 (0.16)
Lys_AID	0.25 (0.11)	0.26 (0.12)	1.04 (0.16)
His_AID	0.17 (0.10)	0.17 (0.10)	1.03 (0.14)
Arg_AID	0.18 (0.10)	0.19 (0.11)	1.03 (0.15)
Pro_AID	0.19 (0.10)	0.20 (0.11)	1.03 (0.15)

** The correlation is significant.

Asp_AID, aspartic digestibility of terminal ileum; Thr_AID, threonine digestibility of terminal ileum; Ser_AID, serine digestibility of terminal ileum; Glu_AID, glutamic acid digestibility of terminal ileum; Gly_AID, glycine digestibility of terminal ileum; Ala_AID, alanine digestibility of terminal ileum; Val_AID, valine digestibility of terminal ileum; Ile_AID, isoleucine digestibility of terminal ileum; Leu_AID, leucine digestibility of terminal ileum; Tyr_AID, tyrosine digestibility of terminal ileum; Phe_AID, phenylalanine digestibility of terminal ileum; Lys_AID, lysine digestibility of terminal ileum; His_AID, histidine digestibility of terminal ileum; Arg_AID, argnine digestibility of terminal ileum; Pro_AID, proline digestibility of terminal ileum.

**Table 4:** Genetic correlations among fifteen terminal ileal amino acid digestibility traits in crossbred pigs, along with their standard errors (SE)

Trait (%)	Asp_AID(%)	Thr_AID(%)	Ser_AID(%)	Glu_AID(%)	Gly_AID(%)	Ala_AID(%)	Val_AID(%)	Ile_AID(%)	Leu_AID(%)	Tyr_AID(%)	Phe_AID(%)	Lys_AID(%)	His_AID(%)	Arg_AID(%)
Thr_AID	0.04 (0.40)													
Ser_AID	0.99 (0.01)	0.02 (0.42)												
Glu_AID	0.98 (0.01)	0.04 (0.40)	1.00 (0.01)											
Gly_AID	1.00 (1.08)	0.76 (10.22	0.92 (1.28)	0.98 (1.03)										
Ala_AID	0.82 (0.12)	0.18 (0.47)	0.78 (0.14)	0.82 (0.10)	0.26 (1.88)									
Val_AID	0.99 (0.01)	−0.03 (0.41)	0.18 (0.47)	1.00 (0.01)	0.86 (1.38)	0.82 (0.12)								
Ile_AID	1.00 (0.01)	0.10 (0.45)	0.99 (0.01)	1.00 (0.02)	0.90(1.18)	0.86 (0.11)	1.00(0.01)							
Leu_AID	0.95 (0.03)	0.12 (0.43)	0.96 (0.03)	0.96 (0.03)	0.57(1.69)	0.91 (0.07)	0.96(0.03)	0.97 (0.02)						
Tyr_AID	0.96 (0.03)	0.25 (0.50)	0.95 (0.04)	0.97 (0.03)	1.00(1.10)	0.92 (0.07)	0.99(0.02)	0.99 (0.01)	0.99 (0.01)					
Phe_AID	0.93 (0.04)	0.22 (0.36)	0.93 (0.04)	0.94 (0.04)	1.00(1.14)	0.98 (0.03)	0.96(0.03)	0.99 (0.02)	1.00 (0.01)	0.98 (0.02)				
Lys_AID	0.99 (0.01)	0.24 (0.42)	0.99 (0.01)	0.99 (0.01)	1.00(1.25)	0.82 (0.11)	0.99(0.01)	0.96 (0.03)	0.96 (0.03)	0.93 (0.05)	0.92 (0.05)			
His_AID	0.59 (0.32)	−0.20 (0.38)	0.42 (0.36)	0.41 (0.34)	1.00(1.10)	1.00 (1.25)	0.50(0.34)	0.48 (0.37)	0.46 (0.35)	0.59 (0.41)	0.54 (0.29)	0.54 (0.34)		
Arg_AID	0.12 (0.42)	0.01 (0.46)	−0.11 (0.46)	0.22 (0.42)	1.00(1.39)	0.04 (0.52)	−0.12 (0.44)	−0.10 (0.49)	0.06 (0.46)	0.03 (0.53)	0.23 (0.37)	0.24 (0.44)	0.47 (0.39)	
Pro_AID	−0.09 (0.47)	−0.06 (0.48)	−0.23 (0.51)	−0.18 (0.48)	1.00 (7.53)	−0.30 (0.63)	−0.18 (0.48)	−0.29 (0.54)	−0.44 (0.60)	−0.27 (0.65)	−0.12 (0.44)	−0.29 (0.52)	0.85 (0.21)	−0.12 (0.54)

Asp_AID, aspartic digestibility of terminal ileum; Thr_AID, threonine digestibility of terminal ileum; Ser_AID, serine digestibility of terminal ileum; Glu_AID, glutamic acid digestibility of terminal ileum; Gly_AID, glycine digestibility of terminal ileum; Ala_AID, alanine digestibility of terminal ileum; Val_AID, valine digestibility of terminal ileum; Ile_AID, isoleucine digestibility of terminal ileum; Leu_AID, leucine digestibility of terminal ileum; Tyr_AID, tyrosine digestibility of terminal ileum; Phe_AID, phenylalanine digestibility of terminal ileum; Lys_AID, lysine digestibility of terminal ileum; His_AID, histidine digestibility of terminal ileum; Arg_AID, argnine digestibility of terminal ileum; Pro_AID, proline digestibility of terminal ileum.

**Table 5:** Distribution of SNPs after quality control and the average distances between SNPs on each Sus *Scrofa* chromosome^1)^

Chromosome	Chromosome length (Mb)	Total SNPs	Density of filtered SNPs (kb)
1	315.32	3,791	83.18
2	162.53	2,687	60.49
3	144.35	2,398	60.20
4	143.47	2,450	58.56
5	111.51	1,982	56.26
6	157.73	2,783	56.68
7	134.76	2,360	57.10
8	148.49	2,368	62.71
9	153.66	2,581	59.53
10	79.39	1,348	58.89
11	87.67	1,495	58.64
12	64.02	1,095	58.47
13	218.61	2,854	76.60
14	153.84	2,670	57.62
15	157.65	2,336	67.49
16	86.88	1,486	58.46
17	69.28	1,173	59.06
18	61.20	1,045	58.57
X	144.29	2,517	57.32
Y	1.64	11	148.65
Average	129.81	2,072	83.18

1) SNP density was presented as the average physical distance between two adjacent SNP loci.

SNP, single nucleotide polymorphism.

**Table 6:** SNP sites significantly associated with terminal ileal amino acid digestibility

No.	Traits	SNP ID	Chr	Pos (bp)^1)^	REF	ALT	Effects^2)^	SE	p-value^3)^	λ-value
1	Asp_AID.GLM	WU_10.2_3_33019982	3	33019982	C	T	0.357632402	0.071283765	7.59E-07	67.56
2	Leu_AID.FarmCPU	WU_10.2_3_33019982	3	33019982	C	T	0.356979948	0.070632704	6.31E-07	68.83
3	Leu_AID.GLM	WU_10.2_3_33019982	3	33019982	C	T	0.366333936	0.071298352	4.16E-07	73.66
4	His_AID.FarmCPU	WU_10.2_3_33019982	3	33019982	C	T	0.354083167	0.070583726	7.60E-07	67.54
5	His_AID.GLM	WU_10.2_3_33019982	3	33019982	C	T	0.364631706	0.071219167	4.56E-07	72.56
6	Arg_AID.GLM	WU_10.2_3_33019982	3	33019982	C	T	0.385919375	0.0717588	1.22E-07	75.86
7	Pro_AID.GLM	WU_10.2_3_33019982	3	33019982	C	T	0.353382698	0.071386251	1.05E-06	65.97
8	Arg_AID.FarmCPU	WU_10.2_3_33019982	3	33019982	C	T	0.386334891	0.059603975	2.10E-11	106.63
9	Arg_AID.FarmCPU	ALGA0094389	17	34715741	G	A	0.292495803	0.059160402	1.04E-07	73.23

1) Positions of the most significant SNP according to the *Sus Scrofa* Build 10.2 assembly.

2) Effects of the allele that increases phenotype value in the two populations.

3) Genome-wide significant associations are underlined.

SNP, single nucleotide polymorphism; SE, Standard error; Asp_AID, aspartic digestibility of terminal ileum; GLM, general linear model; Leu_AID, leucine digestibility of terminal ileum; His_AID, histidine digestibility of terminal ileum; Pro_AID, proline digestibility of terminal ileum; Arg_AID, argnine digestibility of terminal ileum.

**Table 7:** SNP locus information related to each trait (within 100 kb)

Traits	SNP		Chr	Mapinfo (bp)	Nearby genes^1)^	*Distance* (bp)
Asp; Leu; His; Pro; Arg_AID	WU_10.2_3_33019982	rs329338310	3	33,019,982	TVP23A (ENSSSCG0000002710^1)^	Intron: 2 of 7
Arg_AID	ALGA0094389	rs80818921	17	34,715,741	SynDIG1 (ENSSSCG00000007130)	Intron: 1 of 1

1) Annotated genes nearest to the most significant SNP, gene names starting with ENSSSCG follow the Ensembl nomenclature.

SNP, single nucleotide polymorphism; Asp_AID, aspartic digestibility of terminal ileum; Leu_AID, leucine digestibility of terminal ileum; His_AID, histidine digestibility of terminal ileum; Pro_AID, proline digestibility of terminal ileum; Arg_AID, argnine digestibility of terminal ileum.
